# Feeding and eating disorders in the DSM-5 era: a systematic review of prevalence rates in non-clinical male and female samples

**DOI:** 10.1186/s40337-017-0186-7

**Published:** 2017-12-28

**Authors:** Camilla Lindvall Dahlgren, Line Wisting, Øyvind Rø

**Affiliations:** 1 Regional Department for Eating Disorders, Division of Mental Health and Addiction, Oslo University Hospital, Ullevål HF, Postboks 4950 Nydalen, 0424 Oslo, Norway; 2 Institute of Clinical Medicine, University of Oslo, P.O. Box 1171 Blindern, 0318 Oslo, Norway

**Keywords:** Epidemiology, Prevalence, Eating disorders, DSM-5, Systematic review

## Abstract

**Objective:**

The objective of this study was to systematically review the literature on the prevalence of eating disorders (EDs) during the DSM-5 era, and to report rates of point- and lifetime prevalence.

**Method:**

A PubMed search was conducted targeting articles on the epidemiology of EDs, in particular, reported rates of prevalence. The review was performed in accordance with PRISMA guidelines, and was limited to DSM-5 based eating disorder diagnoses published between 2012 and 2017.

**Results:**

A total of 19 studies fulfilled inclusion criteria and were included in the study.

**Discussion:**

Following the transition to DSM-5, it is evident that the prevalence of eating disorder not otherwise specified (EDNOS)/other specified feeding and eating disorders (OSFED) has decreased as intended, and there is preliminary evidence suggesting that rates of anorexia nervosa (AN) and bulimia nervosa (BN) and binge eating disorder (BED) have increased. Further, we observed higher rates of BED prevalence among females compared to males, with rates increasing with age. A limitation to the study was the search date, and that none of the included studies investigated the “new” DSM-5 feeding disorders avoidant restrictive food intake disorder (ARFID), pica or rumination disorder warranting attention in future studies investigating the epidemiology of feeding and eating disorders.

## Plain English summary

The aim of writing this literature review was to provide the reader with an overview of published studies using the newest version of the Diagnostic and Statistical Manual of Mental Disorders, the DSM-5, in assessing eating disorder prevalence. A literature search was performed in accordance with well established guidelines, resulting in 19 studies fulfilling inclusion criteria. As intended, rates of the residual eating disorder category “other specified feeding and eating disorders” had increased, and preliminary evidence supported increased prevalence of anorexia nervosa, bulimia nervosa and binge eating disorder. No studies assessing the prevalence of feeding disorders (i.e. Pica, Rumination Disorder and Avoidant Restrictive Food Intake Disorders) were identified.

## Background

In 2013, the Diagnostic and Statistical Manual of Mental Disorders, Fourth Edition (DSM-IV) [1] was replaced by its successor, the DSM-5 [2], yielding a number of adjustments in diagnostic criteria across psychiatric diagnoses. For eating disorders (ED) in specific, a main intention of the DSM-5 adjustments was to decrease the number of ED cases falling into the former diagnostic category “eating disorder not otherwise specified” (EDNOS), a poorly defined and heterogeneous residual category representing the majority of DSM-IV ED cases. This was done by removing binge eating disorder (BED) from the DSM-IV EDNOS category, and reintroducing it as an independent and specified DSM-5 diagnosis, and by expanding the boundaries of anorexia nervosa (AN) and bulimia nervosa (BN). The DSM-5 retained practically all core AN features, but clarified the weight criteria by changing the wording from “a body weight less than 85% of that expected” to “significantly low weight”. Also, the amenorrhea criterion was removed. For BN, the minimum frequency of binge eating episodes and inappropriate compensatory behavior was reduced from twice a week to once a week. In addition to these changes, three disorders previously reserved for children and classified as ‘Feeding and Eating Disorders of Infancy or Early Childhood’ were revised and introduced in the DSM-5 as independent diagnostic categories: pica, avoidant/restrictive food intake disorder (ARFID) and rumination disorder. Finally, EDNOS was replaced by other specified feeding and eating disorders (OSFED) including atypical AN, subthreshold BN and subthreshold BED, purging disorder (PD), night eating syndrome (NES), as well as unspecified feeding and eating disorders (UFED) representing cases where behaviors cause clinically significant distress/impairment of functioning, but fail to meet full criteria for a feeding or eating disorder.

In the years to come, the new diagnostic criteria for EDs are likely to yield alterations in reported point (proportion of individuals affected by a disorder at the one specific point in time) and lifetime (proportion of individuals having been affected by a disorder at any time in life up to the measurement point) prevalence rates. Historically, ED prevalence rates reported using the DSM-IV have varied considerably across studies, with discrepancies being a result of a number of methodological issues such as inconsistent use of assessment instruments [3], variations in study designs [4] and estimates calculated using non-representative samples [5]. It is therefore important to assess trends in occurrence rates on a regular basis, taking into account changes to diagnostic criteria as well as methodological aspects influencing observed trends.

Following the transition from DSM-IV to DSM-5, it is relevant to review whether the revisions to the DSM-IV have resulted in alterations in diagnostic distribution, and to identify diagnostic categories in need of further research. Four years into the DSM-5 era, a small number of studies do, in fact, show that the new diagnostic system alters the representation and distribution of ED diagnoses with evidence of increased lifetime prevalence of BN and BED [6] and increased lifetime [7] and point prevalence [8] of AN. Further, reduced numbers of identified OSFED and UFED cases [9–11] have also been reported. However, only a limited number of studies have sought to investigate the prevalence of DSM-5 feeding disorders (i.e. pica, ARFID and rumination disorder), or the occurrence of the OSFED categories such as subclinical AN, BN and BED, as well as NES and PD. In sum, there have been important recent developments that warrant further attention. These includes the emergence of new empirical findings from studies assessing ED prevalence using DSM-5 criteria, as well as findings from retrospective studies recoding DSM-IV diagnoses into new DSM-5 ED categories. The aim of this study was to systematically review the field for studies reporting ED prevalence based on DSM-5 criteria in non-clinical female and male samples. The review was performed according to PRISMA guidelines [12] and synthesized studies published between 2012 and 2017.

## Methods

### Search strategy

**Fig. 1:**
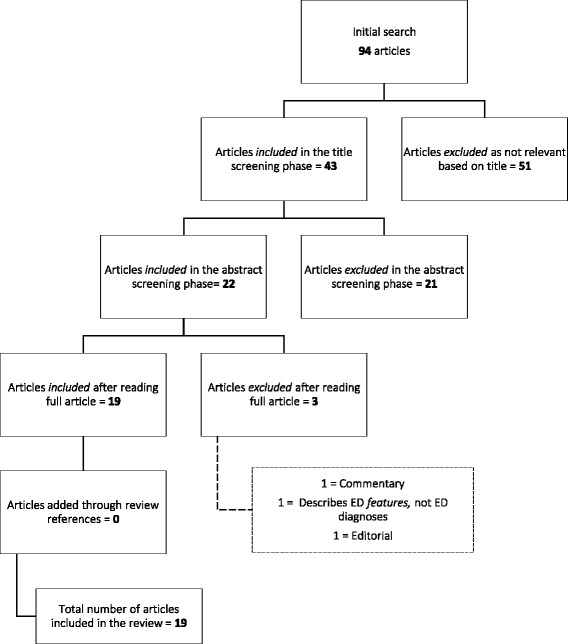
The literature screening process according to PRISMA guidelines

The literature was reviewed in February 2017, using the PubMed search below. The screening process was conducted according to the criteria outlined by the PRISMA guidelines, and is presented in Fig. 1.

(((((((anorexia[All Fields] AND nervosa[All Fields]) AND DSM-5[All Fields] AND prevalence[All Fields]) OR ((bulimia[All Fields] AND nervosa[All Fields]) AND DSM-5[All Fields] AND prevalence[All Fields])) OR ((binge[All Fields] AND eating[All Fields] AND disorder[All Fields]) AND DSM-5[All Fields] AND prevalence[All Fields])) OR ((eating[All Fields] AND disorder[All Fields]) AND DSM-5[All Fields] AND prevalence[All Fields])) OR (OSFED[All Fields] AND DSM-5[All Fields] AND prevalence[All Fields])) OR (PICA[All Fields] AND DSM-5[All Fields] AND prevalence[All Fields])) OR (avoidant[All Fields] AND restrictive[All Fields] AND food[All Fields] AND intake[All Fields] AND disorder[All Fields]) AND DSM-5[All Fields] AND prevalence[All Fields]) OR (rumination[All Fields] AND disorder[All Fields]) AND DSM-5[All Fields] AND prevalence[All Fields])

### Eligibility criteria

Publications were targeted that examined the epidemiology of EDs, in particular ED prevalence rates, in non-clinical samples, and were selected for review according to the criteria outlined below.

A. Articles that presented ED prevalence rates based on DSM-5 criteria (articles presenting recoded diagnostic categories (e.g. from DSM-IV to DSM-5) were also eligible)
B. Articles that were written in English or had an available published English translation
C. Articles that were published in peer reviewed journals

### Exclusion criteria

All papers investigating ED prevalence in clinical samples were excluded. Editorials, commentaries, [systematic] reviews, as well as articles reporting prevalence of disordered eating without utilizing DSM-5 ED diagnoses (e.g. ED *features* or *syndromes*) were omitted. Exclusions were tracked and registered (see Fig. 1). Omitted review articles were screened for additional references. No exclusions for age, gender or geographic areas were applied.

### Title, abstract and article selection

Publication titles in studies obtained through the search strategy outlined earlier were reviewed by the authors CLD and LW. All titles clearly irrelevant to the aim and scope of the paper (e.g. “Food addiction – substance use disorder or behavioral addiction”) were excluded during this initial screening. Publications with ambiguous titles (e.g. “Eating disorders in older women”) and publication with titles clearly relevant to the aim of the paper (e.g. “Prevalence and severity of DSM-5 eating disorders in a community cohort of adolescents”) were included at this stage of the screening process. Abstracts were then screened by CLD and LW using a similar strategy (i.e. screening for irrelevance, ambiguity and certainty), after which full texts were retrieved and assessed for eligibility by CLD and LW using the outlined eligibility and exclusion criteria. Agreement was reached for all included and excluded publications. None of the reviewers were blind to the authors of the texts, nor their affiliations.

## Results

### Search summary

The PubMed search strategy produced 94 titles, which after the initial title screen was reduced to 43. Of these 43, 21 were omitted in the abstract screening process. A total of 22 papers met eligibility criteria and were retained. Subsequently, three full-text studies were excluded based on lack of relevance, leading to a total inclusion of 19 studies. No additional studies were found through the screening of review articles. A more detailed overview of the screening process is presented in Fig. 1 **.**

Table 1 summarizes all included studies. No studies meeting eligibility criteria were published before 2012. Assessments of the following full- and subthreshold diagnoses were reported: ED, AN, BN, BED, OSFED and UFED. OSFED included atypical AN (OSFED-AN) subthreshold BN (OSFED-BN) and subthreshold BED (OSFED-BED), as well as purging disorder (OSFED-PD) and night eating syndrome (OSFED-NES). No studies reporting ARFID, rumination disorder or Pica were identified.

**Table 1:** Overview of included studies published 2012-2017. Studies are grouped by design and listed in chronological order

Author (Year)	N	Country	Gender (N or %)	Age range Mean (*SD*)	Sample	Assessment	Prevalence type	Prevalence (%) of ED, AN, BN (95% CI)	Prevalence (%) of BED, OSFED, UFED (95% CI)
2-STAGE DESIGN									
Mustelin et al. [10]^a^	2825	Finland	♀	22-27 24.4 (0.9)	FinnTwin16 sample	*Screening:* Self-report questionnaire developed for the study and three subscales from the EDI-2 *Diagnosis:* SCID-I/NP	Lifetime	NR	OSFED + UFED: 1.5 (1.1-2.1) OSFED: 0.6 (0.3-1.0) UFED: 0.9 (0.6-1-4)
Mustelin et al. [7]^b^	2825	Finland	♀	22-27 24.4 (0.9)	FinnTwin16 sample	*Screening:* Self-report questionnaire developed for the study and three subscales from the EDI-2 *Diagnosis:* SCID-I/NP	Lifetime	AN: 3.6 (2.7-4.2)	NR
Solmi et al. [16]^c^	1698	UK	♀ (66%) ♂ (44%)	16-90 (mean and SD not available)	SELCoH	*Screening:* SCOFF *Diagnosis:* SCID-I-N/P	Point	ED: 7.4 (4.1-)13.0 AN: 0.0 (0) BN: 0.8 (0.4-1.9)	BED: 3.6 (1.4-9.0) OSFED: 2.4 (0.9-6.7) OSFED-PD: 0.6 (0.2-1.5%)
Smink et al. [15]^d^	1597	Holland	♀ (53.9%) ♂ (56.1%)	19.1 (0.6) (age range not available)	Community cohort	*Screening*: Questionnaire on mental health and social functioning, height/weight and WHO-CIDI *Diagnosis*: SCID-I and parts of EDE	Lifetime (L) Point (P)	♀ ED (L, P): 5.7, 3.7 (4.2-7.5, 2.6-5.2) AN (L, P): 1.7, 1.2 (1.0-2.9, 0.6-2.1) BN (L, P): 0.8, 0.6 (0.3-1.7, 0.2-1.3) ♂ ED (L, P): 1.2, 0.5 (0.6-2.3, 0.1-1.4) AN (L, P): 0.1, 0.1 (0.0-0.8, 0.0-0.8) BN (L, P): 0.1, 0.1 (0.0-0.8, 0.0-0.8)	♀ BED (L, P): 2.3, 1.6 (1.4-3.6, 0.9-2.7) OSFED (L, P): 0.6, 0.3 (0.2-1.3, 0.1-1.0) UFED (L, P): 0.2, 0.0 (0.0-0.8, 0) ♂ BED (L, P): 0.7, 0.3 (0.2-1.6, 0.0-1-0) OSFED (L, P): 0.3, 0.0 (0.0-1.0, 0) UFED (L, P): 0.0, 0.0 (0,0)
Machado et al. [9]	3048	Portugal	♀	12-23 16.2 (1.3) 18-58 21.8 (4.3)	Female high-school and university students	*Screening*: EDE-Q *Diagnosis*: EDE	Point	ED: 3.87 (CI NR) AN: 0.69 (CI NR) BN: 0.59 (CI NR)	BED: 0.62 (CI NR) EDNOS: 1.97 (CI NR)
INTERVIEW									
Mohler-Kuo et al. [4]^e^	10,038	Switzerland	♀ (56%) ♂ (44%)	15-60 (mean and SD not available)	Household survey	WHO-CIDI	Lifetime (L) 12-month (12-m)	♀AN (L, 12-m): 1.9, 0.07 (1.6-2-3, 0.03-0.2) AN ♂ (L, 12-m): 0.2, 0.03 (0.1-0.4, 0.004-0.2)	NR
Hay et al. [11]^f^	6041	Australia	♀ (−) ♂ (−)	15-96 (mean and SD not available)	Cross sectional population sample.	Items based on diagnostic items from the EDE	Point (3-month)	Total sample (% females): ED: 16.3 (15.4-17.3) AN: 0.46 (83%) (0.32-0.67) BN: 0.66 (69%) (0.49-0.9)	Total sample (% females): BED: 5.58 (57%) (5.03-6.19) OSFED-BN: 0.70 (74%) (0.51-0.94) OSFED-BED: 6.92 (55%) (6.31-7.59) OSFED-PD: 0.58 (77%) (0.42-0.80) UFED: 1.41 (73%) (1.14-1.74)
Munn-Chernoff et al. [26]^g^	3230 1790^MZ^ 1440^DZ^	USA	♀	18-29 Median: 22 (mean and SD not available)	Population-based twin study	Adapted version of SSAGA	Lifetime	AN: 1.37 (1.00-1.84)	OSFED-PD: 3.77 (3.14-4.49)
Fairweather-Smith & Wade [19]^h^	699	Australia	♀	12.7-19.8 (across 3 waves) (age range not available)	Adolescent female twin pairs	EDE	Calculated a total prevalence rate based on wave 1-3	ED: 10.4 (8.3-12.9) AN: 2.0 (CI NR) BN: 1.0 (CI NR)	BED: 2.4 (CI NR) OSFED: 5.0 (CI NR) OSFED-AN:1.9 (CI NR) OSFED-BN:2.6 (CI NR) OSFED-PD: 0.6 (CI NR)
Stice et al. [17]^i^	496	USA	♀	Baseline: 12-15 Mean = 13 (SD not available)	Community sample	EDDI	Lifetime (L)	AN: 0.8 (± 0.6) BN: 2.6 (± 1.4)	BED: 3.0 (± 1.3) OSFED: 11.5 (± 2.8) OSFED-AN: 2.8 (±1.5) OSFED-BN: 4.4 (±1.6) OSFED-BED: 3.6 (± 1.5) OSFED-PD: 3.4 (± 1.6)
Hudson et al. [18]^j^	888	USA	♀ (66.4%) ♂ (33.6%)	18-70 46.7 (17.4)	First-degree relatives of probands with or without BED	SCID	Lifetime (L) Point (P)	NR	♀ BED (L, P): 3.6, 1,7 (CI NR) ♂ BED (L, P): 2.1, 0.8 (CI NR)
SELF-REPORT									
Cossrow et al. [27]^k^	22,397	USA	♀ (54.4%) ♂ (45.6%)	≥18 51.1(15.8) (age range not available)	NHWS sample	Questions assessing BED criteria through a self-administered Internet survey	Lifetime (L) 3-month (3-m) 12-month (12-m)	NR	BED ♀ + ♂ (L, 3-m, 12-m): 2.03, 1.19, 1.64 (1.83-2.26, 1.04-1.37, 1.45-1.85) BED ♀: (L, 3-m, 12-m): 2,61, 1,60, 2.00 (2.34-2.92, 1.38-1.85, 1.76-2.28) BED ♂: (L, 3-m, 12-m): 1.41, 0,76, 1,24 (1.13-1.77, 0.56-1.03, 0.97-1.59)
Hammerle et al. [36]^l^	1654	Germany	♀ (*N* = 873) ♂ (*N* = 781)	13.4 (0.8) (age range not available)	National school-based cross-sectional survey	SIAB-S (as questionnaire) and EDI-2	Point	*Full syndrome* (sex ratio female-male)	
								AN: 0.3 (5:0) (0.1-0.7) BN: 0.4 (5:1) (0.2-0.8)	BED: 0.5 (5:3) (0.2-0.9) OSFED-AN: 3.6 (45:13) (2.7-4-5) OSFED-BN:0.0 (−) (0,-) OSFED-BED: 0.0 (−) (0,-) OSFED-PD: 1.9 (22:9) (1.3-2.77)
Flament et al. [14]^m^	3043	Canada	♀ (*N* = 41.2%) ♂ (*N* = 58.8%)	11-21 14.2 (1.6)	Community sample	EDDS	Point	ED (♀, ♂): 4.46, 2.21 (4.4-4.5, 1.5-3.2) AN (♀, ♂): 0.06, 0.0 (0.00-0.31, 0) BN (♀, ♂): 2.01, 1.31 (1.23-3.25, 0.80-2.13)	BED (♀, ♂): 0.68, 0.16 (0.27-1.71, 0.04-0.65) OSFED-PD (♀, ♂): 1.51, 0.74 (0.89-2.53, 0.38-1.41)
Flament et al. [13]^n^	3022	Canada	♀ (*N* = 1789) ♂ (*N* = 1233)	11-20 14.2 (1.6)	Community sample	EDDS	Point	ED (♀ + ♂): 3.7 (2.8-4.7) AN (♀ + ♂): 0.1 (0.0-0.1) BN (♀ + ♂): 1.6 (1.61.1-2.5)	BED (♀ + ♂): 0.5 (0.2-1.2) OSFED-PD (♀ + ♂): 1.4 (1.0-1.9)
de Zwaan et al. [21]^o^	2460	Germany	♀ (51.1%) ♂ (48.9%)	14-85 48.1 (19.0)	Population sample	NEQ and EDE-Q8	Point	NR	OSFED-NES: 1.1 (CI NR)
Runfola et al. [22]	1636	USA	♀ (59.5%) ♂ (40.5%)	18-26 20.9 (1.7)	University students	An online survey using the NEQ	Point	NR	OSFED-NES: 4.2 (CI NR)
Allen et al. [20]^p^	1383	Australia	♀ (49%) ♂ (51%)	14-20 14-years: 14.0 (0.2) 17-years: 16.9 (0.2) 20-years: 20.0 (0.4)	Prospective, population-based cohort study	Items adapted from the ChEDE and the EDE-Q	Point	ED (♀, ♂) 14-yrs: 8.5, 1.2 17-yrs: 15.2, 2.6 20-yrs: 15.2, 2.9 AN (♀, ♂) 14-yrs: 0.3, 0.0 17-yrs: 1.4, 0.0 20-yrs: 0.6, 0.0 BN (♀, ♂) 14-yrs: 2.7, 0.4 17-yrs: 8.7, 0.7 20-yrs: 7.9, 1.6	BED (♀, ♂) 14-yrs: 1.8, 0.0 17-yrs: 1.4, 1.2 20-yrs: 4.1, 0.7 OSFED (♀, ♂) 14-yrs: 3.6, 0.7 17-yrs: 4.1, 0.9 20-yrs: 2.7, 0.6 OSFED-PD (♀, ♂) 14-yrs: 2.7, 0.4 17-yrs: 2.1, 0.6 20-yrs: 1.6, 0.3 OSFED-AN (♀, ♂) 14-yrs: 0.9, 0.3 17-yrs: 0.0, 0.0 20-yrs: 0.1, 0.3
OTHER									
Trace et al. [6]^q^	13,295	Sweden	♀	20-47 (mean and SD not available)	Twin registry subsample	An expanded SCID based instrument	Lifetime	BN: 1.6 (CI NR)	BED 0.4 (CI NR)

*Note.* Prevalence rates are presented exactly as reported in their respective studies. ♀ = Females; ♂ = Males

Abbreviations: *95% CI* 95% Confidence Interval, *NR* Not Reported, *ED* Eating Disorders, *AN* Anorexia Nervosa, *ARFID* Avoidant Restrictive Food Intake Disorder, *BN* Bulimia Nervosa, *BED* Binge Eating Disorder, *OSFED* Other Specified Feeding and Eating Disorders, *UFED* Unspecified Feeding and Eating Disorders, *L* Lifetime prevalence, *P* Point prevalence, *12-m* 12-month prevalence, *3-m* 3-month prevalence

^a^ = Prevalence rates are based on the same sample as in Mustelin et al. [7]^b^. Reported prevalence rates are based on observed cases. Finn Twin16 is a nationwide population based cohort. SCID I-N/P = Structured Clinical Interview for DSM-IV-TR Axis I Disorders, Research Version, Non-Patient Edition [37]; EDI-2 = Eating Disorder Inventory-2 [38]

^b^ = Prevalence rates are based on the same sample as in Mustelin et al. [10]^a^. AN diagnoses were first assessed using DSM-IV, then retrospectively recoded using DSM-5 criteria

^c^ = SELCoH = South East London Community Health Study. SCOFF = Sick, Control, One stone, Fat, Food Questionnaire [39]

^c^ = SCID-I = Structured Clinical Interview for DSM-IV Axis I Disorders

^d^ = WHO-CIDI = World Health Organization Composite International Diagnostic Interview [40]

^e^ = EDE = the Eating Disorder Examination [41]

^g^ = MZ = Monozygotic twins; DZ = Dizygotic twins; SSAGA = An adaptation of the Semi-Structured Assessment on the Genetics of Alcoholism [42];

^h^ = CI was not reported (NR) for separate ED diagnoses

^i^ = EDDI = The Eating Disorder Diagnostic Interview [43]; The diagnostic category “Feeding or eating disorder not elsewhere classified (FEDNEC)” was renamed by the authors of the current review, to OSFED

^j^ = BED diagnoses were first assessed using DSM-IV, then retrospectively recoded using DSM-5 criteria. CI was not reported (NR) for any ED diagnoses

^k^ = NHWS = the National Health and Wellness Survey

^l^ = SIAB-S = the Structured Interview for Anorexia and Bulimia Nervosa Self-report [44]; OSFED-AN = Atypical AN (all criteria is med except significantly low weight), OSFED-BN = Subthreshold BN (of low frequency and/or limited duration), OSFED-BED = Subthreshold BED (of low frequency and/or limited duration), OSFED-PD = Purging Disorder (Recurrent purging in the absence of binge eating)

^m^ = EDDS = the Eating Disorder Diagnostic Scale [45]. NB: Prevalence rates originate from the Flament et al. [14]^m^ study where overall (♀ + ♂) ED rates are reported

^n^ = Prevalence rates based on the same sample, stratified by gender are presented in Flament et al. [14]^n^

^o^ = NEQ = Night Eating Questionnaire [23]; EDE-Q8 = The Eating Disorder Examination Questionnaire 8 [24]

^p^ = ChEDE = the Child Eating Disorder Examination [46]. Confidence intervals were reported visually (using error bars) in the study, but were not presented clearly enough to exclude risk of misinterpretation, and were therefore not included in the table

^q^ = Prevalence rates reported represent binge eating frequency per month ≥4 times

The majority of included studies presented data from US samples (*N* = 5), closely followed by Australia (*N* = 3), Germany (*N* = 2), Finland (N = 2) and Canada (N = 2). Portugal, Sweden, United Kingdom, Switzerland and the Netherlands all represented one individual study. Ages ranged from 11 to 96, with eight studies reporting data from adults only (>18), nine studies reporting rates from mixed age samples (i.e. children, adolescents *and* adults) and two studies presented data collected in samples below the age of 15. Sample sizes ranged from 496 to 22,397, and approximately 25% (*N* = 5) of the included studies followed a two-stage design approach in estimating ED prevalence. Six studies used interviews alone to establish ED diagnoses, and seven studies employed self-reports. Trace et al. [6] did not specify the design of the study. Disregarding prevalence reports using data from overlapping samples [7, 10, 13, 14], all studies employing 2-stage designs had used different screening instruments.

### Prevalence rates

In 2-stage design studies, lifetime AN prevalence rates in females ranged from 1.7% [15] to 3.6% [9], and point prevalence ranged from 0.67% [9] to 1.2% [15]. Only one study reported male AN prevalence and they found both point and lifetime to be 0.1% [15]. BN was most commonly assessed using point prevalence, but was only assessed in two out the five 2-stage design studies [9, 15]. These studies reported nearly identical point BN prevalence rates of 0.59% and 0.6% respectively in females. Point prevalence rates for BED were reported in three studies, and ranged from 0.62% [9] (females only) to 3.6% [16] (males and females combined). Lifetime prevalence rates for OSFED ranged from 0.3% in males [15] to 0.6% in females [10] and point prevalence from 0.0% in males [15] to 2.4% in a sample combining male and females rates. Lifetime prevalence rates of UFED in females were 0.2% [15] and 0.9% [10] respectively.

In interview based studies, lifetime prevalence of AN in females ranged from 0.8% [17] to 1.9% [4]. Lifetime BN prevalence in females of was only reported in one of these studies, with a rate of 2.6% [17]. In two out of the six interview-based studies, lifetime rates of BED were reported. BED in females were 3.0% [17] and 3.6% [18] respectively, whereas the corresponding rates for males were only assessed in one study [18] reporting a rate of 2.1%. In females prevalence of OSFED was reported in the interview based prevalence studies, with overall OSFED point prevalence rates ranging from 5% [19] to lifetime prevalence rate of 11% [17].

Point prevalence was most commonly reported in studies using the self-report design. Here, AN rates ranged from 0.06% [14] to 1.2% in females [15]. BN prevalence rates ranged from 0.45% [11] to 8.7% in females [20]. BED rates ranged from 0.0% (in males) [20] to 4.1% (in females). Two studies [21, 22] set out to investigate the occurrence of night eating syndrome (NES). In these studies, point prevalence was established using the Night Eating Questionnaire (NEQ) [23] and a brief version of the Eating Disorder Examination Questionnaire, the EDE-Q8 [24]. Rates reported for both genders were 1.1% and 4.2% respectively.

## Discussion

The current study reviewed the prevalence of DSM-5 eating disorders. A total of 19 studies were identified in our literature search, with results showing substantial variability in prevalence rates. A wide range of both point- and lifetime prevalence rates were reported across included studies, with variance being dependent on, first and foremost, the methodologies applied.

As intended, a number of studies now support the decline in EDNOS/OSFED prevalence following the DSM-5 diagnostic revision intention [10, 11]. Decreased EDNOS/OSFED prevalence has also been reported in studies having recoded ED diagnoses [9, 13, 20]. However, although the introduction of the new DSM-5 criteria appears to have resolved some of the challenges associated with EDNOS, OSFED still represents a heterogeneous group in the DSM-5, including a variety of different ED conditions. For example, PD, which has been shown to be associated with significant medical complications [25], was investigated specifically in three studies in our review [11, 14, 26]. Prevalence rates ranged from 0.58% (CI 0.42-0.80) – 3.77% (CI 3.14-4.49) with results illustrating a trend towards higher rates in females compared to males, as well as higher rates in adults compared to adolescents. However, it should be noted that lifetime prevalence rates as opposed to point prevalence rates, in general, is expected to be higher, especially when assessed in older populations. Night eating syndrome (NES) was also included in the DSM-5 OSFED category, and specifically assessed in two of the reviewed studies. [21, 22] Both adopted the self-report measure Night Eating Questionnaire (NEQ) [23]. Whereas de Zwaan and colleagues [23] reported a point prevalence of 1.1% among males and females aged 14-85 years (mean age 48), Runfola et al. [24] reported a prevalence of 4.2 among males and females aged 18-26 years (mean age 21). These discrepant findings in prevalence may relate to the differences in age between the two samples, but more research is warranted to investigate this further. Additionally, the new category Unspecified Feeding or Eating Disorder (UFED) was introduced in the DSM-5. UFED is used to describe symptoms characteristic of feeding or eating disorders causing significant stress or impairments in functioning, but without meeting full criteria for an ED diagnosis. The UFED category can be used when reasons for not meeting full criteria is lacking or not further specified by clinicians. Three of the included studies [10, 11, 15] reported prevalence of UFED, with rates ranging from 0.0% to 1.41%. Further research is needed to yield more information about the prevalence of PD, NES, and UFED.

After being included as an EDNOS in DSM-IV, BED was introduced as a specified and independent diagnostic category in DSM-5. Prevalence of BED was reported in several studies included in this review, and the prevalence of BED generally increased by increasing age in the reviewed studies. For example, Allen et al. [20] assessed BED among 1383 young Australian males and females at ages 14, 17, and 20. Point prevalence increased from 1.8% to 4.1% from age 14 to 20 (increase from 0.0% to 0.7% in males). Similarly, Stice et al. [17] investigated 496 young females (mean age 13 years at baseline), and reported a cumulative incidence of 2.7% and a lifetime prevalence at 3.0% by age 20. Finally, Trace et al. [6] assessed a larger and older sample of 13,295 females aged 20-47 years, with a reported lifetime prevalence of 5.8%. Prevalence of BED was generally higher among females than males across studies. A potential explanation for variability in BED prevalence rates is the inconsistent or lack of use of the DSM-5 marked distress criterion and binge eating specifiers such as guilt after binge eating [11].

### Impact of the DSM-5 on prevalence estimates

It is of interest to review whether the transition from DSM-IV to DSM-5 has yielded the intended alterations in reported prevalence, i.e. increased prevalence of AN and BN, and decreased prevalence of the residual category EDNOS/OSFED. Several of the included studies in the present review have estimated prevalence of EDs as defined by both DSM-IV and DSM-5 in the same sample. Such recoding of diagnostic categories according to DSM-IV versus DSM-5 informs us on the impact of the DSM-5 on different ED prevalence rates. One study [7] assessed prevalence of AN by first adopting DSM-IV criteria. Subsequent to recoding according to the DSM-5 criteria, the authors observed a 60% increase in lifetime prevalence of AN among the 2825 female participants (mean age 24 years), from 2.2 to 3.6%. Another study recoding from DSM-IV to DSM-5 diagnoses investigated 1383 males (49%) and females at ages 14, 17, and 20 [20]. Significantly greater ED prevalence rates were reported when using DSM-5 criteria at all ages for females, and at age 17 only for males. These findings are in line with the study of Flament et al. [13] reporting that prevalence of full-threshold EDs increased from 1.8 to 3.7% after recoding from DSM-IV to DSM-5 criteria. Decreased prevalence of the residual ED categories (EDNOS/OSFED) when adopting DSM-5 criteria have also been reported across studies having recoded ED diagnoses [9, 13, 20]. It is worth noting that although research recoding ED prevalence from DSM-IV to DSM-5 has been important in the initial evaluation of the proposed DSM-5 criteria, these studies may have missed patients who initially did not qualify for an ED diagnoses based on DSM-IV, but who would have met criteria for a DSM-5 ED. This may imply a risk of inaccurate prevalence estimates in these studies.

A main intention of the DSM-IV revisions was to minimize the use of catch-all diagnoses such as Eating Disorders Not Otherwise Specified (EDNOS), the most frequently, reported ED diagnosis in the DSM-IV. However, the revisions made may not only contribute to altered rates of AN, BN and OSFED diagnoses, but may also increase the likelihood of reaching the threshold for a formal ED diagnosis. As the new OSFED category includes disorders which lack strict diagnostic criteria (e.g. PD and NES which lacks frequency criteria), clinicians and researchers should be vigilant with regards to the characteristics, especially significant distress and impairments, separating eating *disturbances* from eating *disorders.*

### Methodological aspects

Methodological aspects are likely to influence both lifetime- and point prevalence rates including assessment measures adopted and samples investigated [3]. The majority of studies in this review adopted self-report to assign ED diagnoses, followed by interviews and 2-stage designs. Although self-report assessments have its obvious advantages in terms of being cost- and time effective, only diagnostic interviews can help determine the presence (or absence) of a formal ED diagnosis as defined by the DSM. Methodological issues such as these should be considered when interpreting prevalence rates across studies. In addition to assessment measure per se, recruitment strategy and design are important aspects to consider when evaluating quality of studies and consequently reliability of results. The 2-stage design, including stage one with screening followed by stage two with clinical diagnostic interview, have been considered the preferred approach to estimate prevalence rates. In the current study, aiming to review the prevalence of diagnosable EDs as defined by the diagnostic manual DSM-5, it may therefore be timely to consider the included 2-stage design studies [7, 9, 10, 15, 16] to be among the highest ranked studies in terms of quality. However, another marker of quality in epidemiological studies is sample size. The reviewed studies with the largest sample sizes include samples of *N* = 6041 [11], 13,295 [6], 10,038 [4], and 22,397 [27]. It is worth noting that none of these latter studies are among the mentioned 2-stage design studies, demonstrating the necessity to consider multiple methodological aspects when evaluating quality. In addition to the above-mentioned aspects, sample characteristics are likely to influence reported prevalence rates, and includes both age ranges and gender distributions. One of the intentions of the DSM-5 was to better capture EDs in males than during the DSM-IV era, and was a central rationale for removing the amenorrhea criteria for AN. However, as the DSM-5 revisions have contributed to higher prevalence of full-threshold AN and BN in general, it is difficult to detect whether the DSM-5 has led to better detection of male EDs per se. Also, although the amenorrhea criterion has been removed, the core ED psychopathology outlined in the DSM-5 is still biased towards females as it focuses on drive for thinness rather than muscularity. Furthermore, assessment measures used to detect ED psychopathology are often gender biased in that they have been developed to capture “female” psychopathology and symptoms, and also, as they most commonly are validated using female samples [28, 29]. Issues such as these may complicate the detection and description of ED pathology in males. Another methodological issue subsequent to the introduction of DSM-5 is the removal of a specific weight threshold for AN. This has clear advantages, maybe in particular in terms of individual and flexible evaluations in clinical settings, but it is worth noting that in research, the lack of an explicit weight criterion may lead to larger weight variations compared to earlier. In general, it is important to strive for consistent use of DSM-5 categories, which will aid the interpretation of prevalence rates across studies.

New diagnostic categories in the DSM-5 include ARFID (problem with eating not related to weight or shape concerns, leading to inability to take in adequate nutrition), pica (recurrent consumption of “nonnutritive, nonfood” items), and rumination disorder (RD; i.e. recurrent, effortless regurgitation of food). Although none of the included studies investigated these full-threshold diagnoses, three studies which were excluded due to their sub-threshold nature, addressed features of ARFID [30], pica and RD [31], reporting frequency numbers ranging from 0 to 3.2%. More research is needed to determine the prevalence of these diagnostic categories.

Finally, this review reports the prevalence of eating disorders according DSM-5 criteria. In a historical perspective there have been important changes to the diagnostic criteria since AN and BN were introduced in the DSM-III [32] in 1980. For example, in the DSM-III, the weight loss criteria for AN was “*25% below original body weight*”, which was then revised to “*body weight less than 85% of that expected*” in the DSM-IV [1], and subsequently, redefined in the DSM-5 [2] to “*significantly low body weight*”. For BN there were no criteria for frequency of binge eating and compensatory behavior in the DSM-III. With the DSM-III-R update, the frequency was specified to twice a week, and in DSM-5, it was reduced to once a week. These revisions will greatly influence prevalence rates in the years to come, and are crucial to address when comparing DSM-5 based prevalence to earlier epidemiological studies in EDs.

### Strengths and limitations

This is the first systematic review of DSM-5 prevalence studies in EDs, and offers the reader an early snapshot of the extant prevalence literature. The core strength of the study is the thoroughness of the systematic literature review, and the detailed screening process conducted by two of the authors. Conversely, only one database was used to search the literature and only articles that were written in English (or had an available published English translation) were reviewed representing a limitation of the current study. Further, a meta-analysis was not performed which also represents a potential weakness. In addition, our search date (February 2017) dates our paper. In this interim, three publications relevant to the scope of our paper have been published. Hay et al. [33] investigated the prevalence and burden of ARFID and other DSM-5 EDs in an Australian population, Ernst et al. [34] explored how the DSM-5 revisions affected the prevalence, sex ratio and diagnostic distribution of EDNOS/OSFED in a student sample, and Micali et al. [35] investigated lifetime and 12-month prevalence of EDs amongst women in mid-life. Their relevance to the extant literature warrant a more detailed account in future epidemiological ED studies.

## Conclusions

In conclusion, prevalence rates varied significantly across the 19 studies reviewed, much likely due to variations in study design, diagnostic assessment routines, samples sizes and characteristics. None of the included studies investigated the prevalence of the DSM-5 feeding disorders pica, ARFID and RD, warranting further investigation in future epidemiological studies. As our review is limited by a small number of studies published during a limited time frame, and in addition, fails to capture the full range of DSM-5 diagnoses, reported trends should be interpreted with caution.

## Abbreviations

12-m: 12-month
3-m: 3-month
AN: Anorexia nervosa
ARFID: Avoidant restrictive food intake disorder
BED: Binge eating disorder
BN: Bulimia nervosa
CI: Confidence interval
DSM-5: Diagnostic and statistical manual of mental disorders, fifth edition
DSM-IV: Diagnostic and statistical manual of mental disorders, fourth edition
ED: Eating disorder
EDE: Eating disorder examination
EDI-2: Eating disorder inventory
EDNOS: Eating disorder not otherwise specified
L: Lifetime
NEQ: Night eating questionnaire
NES: Night eating syndrome
NR: Not reported
OSFED: Other specified feeding and eating disorders
P: Point
PD: Purging disorder
SCID I-N/P: Structured clinical interview for DSM-IV-TR axis I disorders, research version, non-patient edition
SCID-I: Structured clinical interview for DSM-IV axis I disorders
SCOFF: Sick, control, one stone, fat, food questionnaire
SELCoH: South East London Community Health Study
UFED: Unspecified feeding and eating disorder
WHO-CIDI: World Health Organization Composite International Diagnostic Interview

## References

[1] American Psychiatric Association (1994). Diagnostic and statistical manual of mental disorders DSM-IV.

[2] American Psychiatric Association (2013). Diagnostic and statistical manual of mental disorders DSM-5.

[3] Lindvall Dahlgren C, Wisting L (2016). Transitioning from DSM-IV to DSM-5: a systematic review of eating disorder prevalence assessment. Int J Eat Disord.

[4] Mohler-Kuo M (2016). The prevalence, correlates, and help-seeking of eating disorders in Switzerland. Psychol Med.

[5] Hoek HW, Van Hoeken D (2003). Review of the prevalence and incidence of eating disorders. Int J Eat Disord.

[6] Trace SE (2012). Effects of reducing the frequency and duration criteria for binge eating on lifetime prevalence of bulimia nervosa and binge eating disorder: implications for DSM-5. Int J Eat Disord.

[7] Mustelin L (2016). The DSM-5 diagnostic criteria for anorexia nervosa may change its population prevalence and prognostic value. J Psychiatr Res.

[8] Ornstein RM (2013). Distribution of eating disorders in children and adolescents using the proposed DSM-5 criteria for feeding and eating disorders. J Adolesc Health.

[9] Machado PP, Goncalves S, Hoek HW (2013). DSM-5 reduces the proportion of EDNOS cases: evidence from community samples. Int J Eat Disord.

[10] Mustelin L, Lehtokari VL, Keski-Rahkonen A (2016). Other specified and unspecified feeding or eating disorders among women in the community. Int J Eat Disord.

[11] Hay P, Girosi F, Mond J (2015). Prevalence and sociodemographic correlates of DSM-5 eating disorders in the Australian population. J Eat Disord.

[12] Moher D (2015). Preferred reporting items for systematic review and meta-analysis protocols (PRISMA-P) 2015 statement. Syst Rev.

[13] Flament MF (2015). Comparative distribution and validity of DSM-IV and DSM-5 diagnoses of eating disorders in adolescents from the community. Eur Eat Disord Rev.

[14] Flament MF (2015). Weight status and DSM-5 diagnoses of eating disorders in adolescents from the community. J Am Acad Child Adolesc Psychiatry.

[15] Smink FR (2014). Prevalence and severity of DSM-5 eating disorders in a community cohort of adolescents. Int J Eat Disord.

[16] Solmi F (2016). Eating disorders in a multi-ethnic inner-city UK sample: prevalence, comorbidity and service use. Soc Psychiatry Psychiatr Epidemiol.

[17] Stice E, Marti CN, Rohde P (2013). Prevalence, incidence, impairment, and course of the proposed DSM-5 eating disorder diagnoses in an 8-year prospective community study of young women. J Abnorm Psychol.

[18] Hudson JI (2012). By how much will the proposed new DSM-5 criteria increase the prevalence of binge eating disorder?. Int J Eat Disord.

[19] Fairweather-Schmidt AK, Wade TD (2014). DSM-5 eating disorders and other specified eating and feeding disorders: is there a meaningful differentiation?. Int J Eat Disord.

[20] Allen KL (2013). DSM-IV-TR and DSM-5 eating disorders in adolescents: prevalence, stability, and psychosocial correlates in a population-based sample of male and female adolescents. J Abnorm Psychol.

[21] De Zwaan M (2014). Prevalence and correlates of night eating in the German general population. PLoS One.

[22] Runfola CD (2014). Prevalence and clinical significance of night eating syndrome in university students. J Adolesc Health.

[23] Allison KC (2008). The night eating questionnaire (NEQ): psychometric properties of a measure of severity of the night eating syndrome. Eat Behav.

[24] Kliem S (2016). The eating disorder examination-questionnaire 8: a brief measure of eating disorder psychopathology (EDE-Q8). Int J Eat Disord.

[25] Forney KJ (2016). The medical complications associated with purging. Int J Eat Disord.

[26] Munn-Chernoff MA (2015). Prevalence of and familial influences on purging disorder in a community sample of female twins. Int J Eat Disord.

[27] Cossrow N (2016). Estimating the prevalence of binge eating disorder in a community sample from the United States: comparing DSM-IV-TR and DSM-5 criteria. J Clin Psychiatry.

[28] Darcy AM (2012). The eating disorders examination in adolescent males with anorexia nervosa: how does it compare to adolescent females?. Int. J. Eat. Disord..

[29] Stanford SC, Lemberg R (2012). Measuring eating disorders in men: development of the eating disorder assessment for men (EDAM). Eat Disord.

[30] Kurz S (2015). Early-onset restrictive eating disturbances in primary school boys and girls. Eur Child Adolesc Psychiatry.

[31] Delaney CB (2015). Pica and rumination behavior among individuals seeking treatment for eating disorders or obesity. Int J Eat Disord.

[32] American Psychiatric Association (1980). Diagnostical and statistical manual of mental disorders.

[33] Hay P (2017). Burden and health-related quality of life of eating disorders, including avoidant/restrictive food intake disorder (ARFID), in the Australian population. J Eat Disord.

[34] Ernst V, Burger A, Hammerle F (2017). Prevalence and severity of eating disorders: a comparison of DSM-IV and DSM-5 among German adolescents. Int J Eat Disord.

[35] Micali N (2017). Lifetime and 12-month prevalence of eating disorders amongst women in mid-life: a population-based study of diagnoses and risk factors. BMC Med.

[36] Hammerle F (2016). Thinking dimensional: prevalence of DSM-5 early adolescent full syndrome, partial and subthreshold eating disorders in a cross-sectional survey in German schools. BMJ Open.

[37] First MB (2002). Structured clinical interview for DSM-IV-TR axis I disorders, research version, non-patient edition (SCID-I/NP).

[38] Garner DM (1991). Eating disorder inventory-2 manual. Psychological assessment resources.

[39] Morgan JF, Reid F, Lacey JH (1999). The SCOFF questionnaire: assessment of a new screening tool for eating disorders. BMJ.

[40] Kessler RC, Ustun TB (2004). The World Mental Health (WMH) survey initiative version of the World Health Organization (WHO) Composite International Diagnostic Interview (CIDI). Int J Methods Psychiatr Res.

[41] Fairburn CG, Cooper Z, O'connor M, Fairburn CG (2008). Eating disorder examination (16.0d). Cognitive behavior therapy and eating disorders.

[42] Bucholz KK (1994). A new, semi-structured psychiatric interview for use in genetic linkage studies: a report on the reliability of the SSAGA. J Stud Alcohol.

[43] Stice E (2008). Dissonance and healthy weight eating disorder prevention programs: long-term effects from a randomized efficacy trial. J Consult Clin Psychol.

[44] Fichter MM, Quadflieg N (2000). Comparing self-and expert rating: a self-report screening version (SIAB-S) of the structured interview for anorexic and bulimic syndromes for DSM-IV and ICD-10 (SIAB-EX). Eur Arch Psychiatry Clin Neurosci.

[45] Stice E, Telch CF, Rizvi SL (2000). Development and validation of the eating disorder diagnostic scale: a brief self-report measure of anorexia, bulimia, and binge-eating disorder. Psychol Assess.

[46] Bryant-Waugh RJ (1996). The use of the eating disorder examination with children: a pilot study. Int. J. Eat. Disord..

